# RVMAB: Using the Relevance Vector Machine Model Combined with Average Blocks to Predict the Interactions of Proteins from Protein Sequences

**DOI:** 10.3390/ijms17050757

**Published:** 2016-05-18

**Authors:** Ji-Yong An, Zhu-Hong You, Fan-Rong Meng, Shu-Juan Xu, Yin Wang

**Affiliations:** School of Computer Science and Technology, China University of Mining and Technology, Xuzhou 21116, China; ajy@cumt.edu.cn (J.-Y.A.); ajysjm@163.com (S.-J.X.); yinwang@cumt.edu.cn (Y.W.)

**Keywords:** relevance vector machine, average blocks, PSSM, protein sequence

## Abstract

Protein-Protein Interactions (PPIs) play essential roles in most cellular processes. Knowledge of PPIs is becoming increasingly more important, which has prompted the development of technologies that are capable of discovering large-scale PPIs. Although many high-throughput biological technologies have been proposed to detect PPIs, there are unavoidable shortcomings, including cost, time intensity, and inherently high false positive and false negative rates. For the sake of these reasons, *in silico* methods are attracting much attention due to their good performances in predicting PPIs. In this paper, we propose a novel computational method known as RVM-AB that combines the Relevance Vector Machine (RVM) model and Average Blocks (AB) to predict PPIs from protein sequences. The main improvements are the results of representing protein sequences using the AB feature representation on a Position Specific Scoring Matrix (PSSM), reducing the influence of noise using a Principal Component Analysis (PCA), and using a Relevance Vector Machine (RVM) based classifier. We performed five-fold cross-validation experiments on yeast and *Helicobacter pylori* datasets, and achieved very high accuracies of 92.98% and 95.58% respectively, which is significantly better than previous works. In addition, we also obtained good prediction accuracies of 88.31%, 89.46%, 91.08%, 91.55%, and 94.81% on other five independent datasets *C. elegans*, *M. musculus*, *H. sapiens*, *H. pylori*, and *E. coli* for cross-species prediction. To further evaluate the proposed method, we compare it with the state-of-the-art support vector machine (SVM) classifier on the yeast dataset. The experimental results demonstrate that our RVM-AB method is obviously better than the SVM-based method. The promising experimental results show the efficiency and simplicity of the proposed method, which can be an automatic decision support tool. To facilitate extensive studies for future proteomics research, we developed a freely available web server called RVMAB-PPI in Hypertext Preprocessor (PHP) for predicting PPIs. The web server including source code and the datasets are available at http://219.219.62.123:8888/ppi_ab/.

## 1. Introduction

Proteins are fundamental molecules of living organisms that participate in nearly all cell functions in an organism. Protein-protein interactions (PPIs) play an essential role in many biological processes. Thus, detection the interactions of proteins become more and more important. Knowledge of PPIs can provide insight into the molecular mechanisms of biological processes, lead to a better understanding of disease mechanisms, and suggest novel methods for practical medical applications. In recent years, a number of high-throughput technologies, such as immunoprecipitation [1], protein chips [2], and yeast two-hybrid screening methods [3,4], have been developed for detecting the large-scale PPIs. However, there are some disadvantages of these experimental approaches, such as time-intensiveness and high cost. In addition, the aforementioned methods suffer from high rates of false positives and false negatives. For these reasons, predicting unknown PPIs is considered a difficult task using only biological experimental methods. Therefore, there is a stronger motivation to exploit computational methods for PPIs.

As a result, a number of computational methods have been proposed to infer PPIs from different sources of information, including tertiary structures, phylogenetic profiles, protein domains, and secondary structures. However, these approaches cannot be employed when prior-knowledge about a protein of interest is not available. With the rapid growth of protein sequence data, the protein sequence-based method is becoming the most widely used tool for predicting PPIs. Consequently, a number of protein sequence-based methods have been developed for predicting PPIs. For example, Martin *et al.* proposed the method that uses a novel descriptor called signature product to predict PPIs [5]. The descriptor is extended to protein pairs by using signature product. The signature product is implemented within a support vector machine (SVM) classifier as a kernel function. Nanni and Lumini [6] used the method based on an ensemble of *K*-local hyperplane distance nearest neighbor (HKNN) classifiers to predict PPIs, where each classifier is trained using a different physicochemical property of the amino acids. Bock and Gough [7] proposed a method that an SVM is used and combined with several structural and physiochemical descriptors to predict PPIs. Chou and Cai [8] used the approach based on the gene ontology and the approach of pseudo-amino acid composition, where a predictor called “GO-PseAA” predictor was established to predict PPIs. Shen *et al*. [9] proposed a method based on a support vector machine (SVM) combined with a kernel function and a conjoint triad feature for describing amino acids to infer human PPIs. Guo *et al.* [10] proposed a sequence-based method that used a support vector machine (SVM) combined with feature representation of auto covariance (AC) descriptor to predict yeast PPIs. Chen *et al.* [11] used a domain-based random forest of decision trees to infer protein interactions. Licamele and Getoor [12] proposed several novel relational features, where they used a Bagging algorithm to predict PPIs. Several other methods based on protein amino acid sequences have been proposed in previous works. In spite of this, there is still space to improve the accuracy and efficiency of the existing methods.

In this paper, a novel computational method was proposed, which can be used to predict PPIs using only protein sequence data. The main aim of this study is to improve the accuracy of predicting PPIs. The main improvements are the results of representing protein sequences using the Average Blocks (AB) feature representation on a Position Specific Scoring Matrix (PSSM), reducing the influence of noise by using a Principal Component Analysis (PCA), and using a Relevance Vector Machine (RVM) based classifier. More specifically, we first represent each protein using a PSSM representation. Then, an Average Blocks (AB) descriptor is employed to capture useful information from each protein PSSM and generate a 400-dimensional feature vector. Next, dimensionality reduction method PCA is used to reduce the dimensions of the AB vector and the influence of noise. Finally, the RVM model is employed as the machine learning approach to carry out classification. The proposed method was executed using two different PPIs datasets: yeast and *Helicobacter pylori*. The experimental results are found to be superior to SVM and other previous methods. In addition, cross-species experiments were also performed on five independent datasets *C. elegans*, *M. musculus*, *H. sapiens*, *H. pylori*, and *E. coli*. Thus, we also obtained good prediction accuracy in the cross-species experiments. The achieved results show that the proposed method is fit for predicting PPIs. These experimental results prove that the proposed method performs incredibly well in predicting PPIs.

## 2. Results and Discussion

### 2.1. Performance of the Proposed Method on Yeast and H. pylori Datasets

In the paper, to prevent the over-fitting of the proposed prediction model and test the reliability of our proposed method, five-fold cross validation was applied in our experiment. More specifically, the whole dataset was divided into five parts; four parts of them were employed for the training model, and one part of them was used for testing. We obtained five models from the yeast and *Helicobacter pylori* datasets by using the above mentioned method, and each model was executed alone in the experiment. In order to ensure fairness, the related parameters of the RVM model were set up the same for the two different datasets, yeast and *Helicobacter pylori*. Here, we selected the “Gaussian” function as the kernel function and choose the following parameters: width = 1 initapla = 1/*N*^2^, and beta = 0, where width represent the width of the kernel function, *N* is the number of training samples, and the value of beta was defined as zero, which represents classification. The experimental results of the prediction models of the RVM classifier combined with Average Blocks and the Position Specific Scoring Matrix and principal component analysis based on the information of protein sequence on yeast and *Helicobacter pylori* datasets are listed in Table 1 and Table 2.

When performing the proposed method on the yeast dataset, we achieved the prediction results of average accuracy, precision, sensitivity, and Matthews’s correlation coefficient (Mcc) of 92.98%, 93.14%, 92.79%, and 86.82%. The standard deviations of these criteria values were 0.99%, 1.00%, 1.2%, and 1.66%, respectively. Similarly, we also gained good prediction results of average accuracy, precision, sensitivity, and MCC of 95.58%, 95.51%, 95.62%, and 91.55% on the *Helicobacter pylori* dataset. The standard deviations of these criteria values were 1.00%, 1.83%, 1.60%, and 1.91%, respectively.

It can be seen from Table 1 and Table 2 that these experiment results demonstrated that the proposed method is accurate, robust, and effective for predicting PPIs. The better performance of prediction PPIs may be attributed to the feature extraction and the choice of classifier of the proposed method. The feature extraction is novel and effective, and the choice of the classifier is accurate. The proposed feature extraction method contains three data processing steps. First, the PSSM matrix not only describes the order information for the protein sequence but also retains sufficient prior information; thus, it is widely used in other proteomics research. As a result, we converted each protein sequence to a PSSM matrix that contains all the useful information from each protein sequence; Second, because Average Blocks method based on the residue conservation tendencies in the same domain family are similar and the locations of domains in the same family are closely related to the length of the sequence, information can be effectively captured from the PSSMs using the Average Blocks method; Finally, while meeting the condition of maintaining the integrity of the information in the PSSM, we reduced the dimensions of each AB vector and reduced the influence of noise using principal component analysis. Consequently, the sample information that was extracted using the proposed feature extraction method is very suitable for predicting PPIs.

### 2.2. Comparison with the Support Vector Machine (SVM)-Based Method

Although our results suggest that the proposed method can obtain good prediction results, to further evaluate the effectiveness of the proposed approach, we compared the prediction accuracy of the proposed method with that of the state-of-the-art support vector machine (SVM) classifier. More specifically, we compared the classification performances between SVM and RVM model on the yeast dataset by using the same feature extraction method. The LIBSVM tool [13] was employed to carry out classification in SVM. A grid search method was used to optimize the corresponding parameters of SVM(c = 0.9, g = 0.6). At the same time, we used a radial basis function as the kernel function in the experiment.

**Figure 1:**
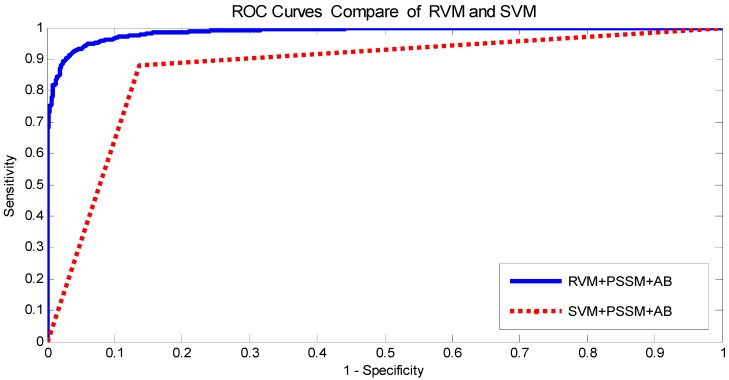
Comparison of Receiver Operating Curve (ROC) curves performed between Relevance Vector Machine (RVM) and support vector machine (SVM) on yeast dataset.

The prediction results of the SVM and RVM methods are summarized in Table 3 on the yeast dataset, and the Receiver Operating Curve (ROC) curves are displayed in Figure 1. From Table 3, the prediction results of the SVM method achieved 85.49% average accuracy, 85.52% average sensitivity, 85.39% average precision, and 75.20% average Mcc, while the prediction results of the RVM method achieved 92.98% average accuracy, 92.79% average sensitivity, 93.14% average precision, and 86.82% average Mcc. The comparison verified that the RVM classifier is significantly better than the SVM classifier. In addition, the ROC curves were analyzed in Figure 1, showing that the ROC curve of the RVM classifier is variously better than that of the SVM classifier. This clearly indicates that the RVM classifier of the proposed method is an accurate and robust classifier for predicting PPIs. There are two possible reasons that the RVM classifier yields significantly better prediction results than the SVM classifier. (1) RVM has a computational advantage that the calculation amount of the kernel function is greatly reduced; (2) RVM overcomes the shortcoming of the kernel function being required to satisfy the condition of Mercer. Because of these reasons, the RVM classifier is significantly better than the SVM classifier. At the same time, we concluded that the proposed method can gain the high prediction accuracy for PPIs.

### 2.3. Performance on Independent Dataset

Since reasonably good prediction results have been yielded by using the proposed method for predicting PPIs, we switched to evaluate its prediction performance on five other independent datasets. It is known that the biological hypothesis of mapping PPIs from one species to another species is that a large number of physically interacting proteins in one organism have “coevolved” so that their respective orthologues in other organisms interact as well. Consequently, we used the train prediction model of the yeast dataset to predict PPIs on five other independent datasets using our prediction model. In the experiment, we selected all 11188 protein pairs of yeast dataset as the training dataset and choose the function “Gauss” as the kernel function and set the optimal parameters “width = 7, initapla = 1/*N*”. We also used the feature extraction based on PSSM combined with Average Blocks and Principal Component Analysis to convert protein pairs of the other five independent datasets into feature vectors that were employed as the testing datasets of RVM classifier. The prediction results of the five cross-species experiments are shown in Table 4. It can be seen from Table 4 that the proposed prediction model obtained good prediction accuracy of 88.31%, 89.46%, 91.08%, 91.55%, and 94.81% on *C. elegans*, *M. musculus*, *H. sapiens*, *H. pylori*, and *E. coli* dataset, respectively. It shows that the Meta model has the ability to predict well the PPIs of five independent datasets with the accuracy of over 88.31%, while the high accuracy of 94.81% has been achieved on *E. coli* dataset.

Interestingly, through these experiment results, it can be proved that the yeast dataset is able to predict the PPIs of other species. In addition, we can find that our prediction model has a strong ability to predict PPIs. The proposed model can be used to discover the organisms whose PPIs data are not available and provided certain assistance for further research.

### 2.4. Comparison with Other Methods

A number of prediction methods based on protein sequences for PPIs have been proposed. To evaluate the effectiveness of our propose method, we compared the prediction ability of our proposed method with existing methods on yeast and *Helicobacter pylori* datasets, respectively. It is shown in Table 5 that five different ways obtained an average prediction accuracy between 75.08% and 89.33% on the yeast dataset, while the proposed method achieved the average prediction accuracy of 95.58%, which obviously higher than that of other five different methods. Similarly, the precision and sensitivity of our proposed method are also superior to those of the other methods. The average prediction accuracy between the five different ways and the proposed method on the *Helicobacter pylori* dataset is displayed in Table 6. From Table 6, we can see that the average prediction accuracies of other five different methods are between 83% and 87.5%. None of these methods obtains higher prediction accuracy than that of 92.68% of our proposed method. It is obvious from Table 5 and Table 6 that the proposed method yielded significantly better prediction results than other existing methods. All these results indicate that the RVM classifier combined with average blocks and the position specific scoring matrix and principal component analysis can improve the prediction accuracy relative to current state-of-the-art methods. Due to using a correct classifier and a novel feature extraction method that captures the useful evolutionary information, thus, the proposed method can gain high prediction accuracy.

## 3. Experimental Section

### 3.1. Dataset

In the paper, we evaluated the proposed method using seven publicly available datasets yeast, *H. pylori*, *C. elegans*, *E. coli*, *H. sapiens*, and *M. musculus.* Yeast and *Helicobacter pylori* are composed of positive datasets and negative datasets, the rest five datasets only consist of positive datasets. All the datasets were obtained from the Database of Interaction Proteins (DIP). In order to better execute the proposed method, 5594 positive protein pairs were selected to build the positive pairs dataset and 5594 negative protein pairs build the negative pairs dataset from the yeast dataset. Similarly, we selected 1458 positive protein pairs to build the positive pairs dataset and 1458 negative protein pairs to build the negative pairs dataset from the *H. pylori* dataset. Consequently, the yeast dataset contains 11,188 protein pairs and the *H. pylori* dataset contains 2916 protein pairs. For the other five independent datasets only contains positive dataset, we selected 4013, 6954, 1420, 1412, and 313 positive protein pairs from *C. elegans*, *E. coli*, *H. pylori*, *H. sapiens*, and *M. musculus*, respectively.

### 3.2. Position Specific Scoring Matrix

Position Specific Scoring Matrix (PSSM) was first used to detect distantly related proteins, which can be created from a set of sequences proteins [19]. A Position Specific Scoring Matrix (PSSM) for a query protein is a M×20 matrix $Y=\left\{Y_{ij}:1=1\ldots M,j=1\ldots 20\right\}$, where *M* is the length of the protein sequence, and 20 represents the 20 amino acids. The PSSM assigns a score $Y_{ij}$ for the jth amino acid in the ith position of the given protein sequence. The score $Y_{ij}$ of the position of a given protein sequence can be expressed as $Y_{ij}=\overset{20}{\underset{k=1}{\sum }}p\left(i,k\right)\times q\left(j,k\right)$, where p(i,k) is the ratio of the frequency of the kth amino acid appearing at position i of the probe to be the total number of probes, and *q*(*j*,*k*) is the value of Dayhoff’s mutation matrix between the jth and *k*th amino acids. As a result, a large score represents a highly conserved position and a small score represents a weakly conserved position.

It is useful for PSSMs to predict protein quaternary structural attributes, disulfide connectivity, and folding patterns [20,21,22,23]. Here, we also use PSSMs to predict PPIs. In this work, we used the Position Specific Iterated BLAST (PSI-BLAST) [24] to build PSSMs for each protein sequence. In order to obtain broadly and highly homologous sequences, the default value of PSI_BLAST were chosen, that is, the e-value parameter was set as 0.001 and three iterations were selected in the proposed method. The resulting PSSMs can be represented as 20-dimensional matrices. Each matrix is composed of *L* × 20 elements, where *L* is the total number of residues in a protein. The rows of the matrix represent the protein residues, and the columns of the matrix represent the 20 amino acids.

### 3.3. Average Blocks

The characteristics of the Average Blocks (AB) was originally described in the literature [25]. Because of each protein has different numbers of amino acids, we cannot directly transform the PSSMs to feature vectors, which will lead to different sizes of feature vectors. To solve the problem, the features are averaged over a local region in PSSMs, which is called as averaged PSSM profile over blocks (Average Blocks), each with 5 percent of a protein sequence. Thus, regardless of the length of a protein sequence, a protein sequence is divided into 20 blocks and every block composes 20 features derived from the 20 columns in PSSMs by using the feature extract method [21]. Related mathematical formula is as follows:

(1) $$\text{AB}\left(k\right)=\frac{20}{N}\overset{\frac{N}{20}}{\underset{p=1}{\sum }}Mt(p+\left(i-1\right)\;\times \;\frac{N}{20},j)\;\;i=1,\ldots ,\;20;j=1,\ldots ,\;20;\;k=j+20\;\times \;\left(i-1\right),$$

where $\frac{N}{20}$ represents the size of the *jth* block, which is 5 percent of the length of a sequence. Where $Mt\left(p+\left(i-1\right)\;\times \;\frac{N}{20},j\right)$ is defined as a 1×20 vector extracted from the PSSM profile at the *ith* positon in the *jth* block. As a result, each sequence has 20 blocks and can be expressed as a 400-dimensional vector by using the method of Average Blocks. The rationale behind Average Blocks is that the residue conservation tendencies in the same domain family are similar, and the locations of domains in the same family are closely related to the length of the sequence [25]. In the paper, finally, each protein sequence of seven datasets was converted into a 400-dimensional vector by using the feature extraction method of Average Blocks.

### 3.4. Principal Component Analysis

Principal component analysis (PCA) is a useful tool to process data. It can analyze the main influencing factors from multiple dimensional datasets and simplify the complex problems. In this way, high-dimensional data can be projected to lower dimensions by computing principal components, which can retain the main information of the original dataset and wipe off the useless information of the original dataset. The useless information called the noise. The basic fundamental of PCA is as follows:

A multivariate dataset can be represented as the following matrix *P*:

(2) $$P=\left(\begin{array}{c} \begin{array}{c} p\left(1\right)\\ \ldots \end{array}\\ p\left(N\right) \end{array}\right),p\left(t\right)=\left[p_{1}\left(t\right)\ldots p_{s}\left(t\right)\right],)t=1,\ldots N)$$

where s is the count of variables, and N is the number of sampling of each variable. PCA is closely related to singular value decomposition (SVD) of matrix and the singular value decomposition of matrix P as follows:

(3) $$P=\overset{s}{\underset{i=1}{\sum }}x_{i}y_{i}z_{i}^{T}$$

where $z_{i}$ represents feature vector of $P^{T}P$ and $y_{i}$ represents feature vector of $PP^{T}$, and $x_{i}$ is a singular value. If there are *m* linear relationships between s variables, then the m singular value is zero. Any row of *P* can be expressed as feature vector $\left(q_{1},q_{2},\ldots ,q_{k}\right)$

(4) $$P^{T}\left(t\right)=\overset{k}{\underset{i=1}{\sum }}x_{i}y_{i}z_{i}=\overset{k}{\underset{i=1}{\sum }}r_{i}\left(t\right)q_{i}$$

where $r_{i}\left(t\right)=p\left(t\right)q_{i}$ is the of projection p(t) on $q_{i}$, feature vector $\left(q_{1},q_{2},\ldots ,q_{k}\right)$ is load vector, and $r_{i}\left(t\right)$ is score.

When there is a certain degree of linear correlation between the variables of matrix, then the projection of final several load vectors of matrix X will be small enough, resulting from measurement noise. As a result, the principal decomposition of matrix X represented by

(5) $$P=r_{1}q_{1}^{T}+r_{2}q_{2}^{T}+\ldots +r_{k}q_{k}^{T}+E$$

where *E* is a noise matrix and can be ignored. This does not result in the obvious loss of useful information of dataset.

As a result, when large multivariate datasets are analyzed, PCA is often desirable to reduce their dimensionality, which can integrate the useful information and reduce the influence of noise for improving the efficiency of data processing. In this paper, in order to reduce the influence of noise and improve the prediction accuracy, we reduce dimensions of the seven datasets from 400 to 350 in the proposed method by using PCA. It can be seen from Table 7 that PCA significantly improves the prediction accuracy by integrating the useful information and reducing the influence of noise.

### 3.5. Relevance Vector Machine

The related theory of the Relevance Vector Machine (RVM) model has been described in detail in the literature [26]. It is assumed that the training sample sets are ${\left\{a_{n},\;t_{n}\right\}}_{n=1}^{N}$ for binary classification problems, $a_{n}\in R^{d}$ is the training sample, $t_{n}\in \left\{0,1\right\}$ represents the training sample label, $t_{i}$ represents the testing sample label, and $t_{i}=b_{i}+\varepsilon_{i}$, where $b_{i}=w^{T}\varphi \left(a_{i}\right)=\overset{N}{\underset{j=1}{\sum }}w_{j}K\left(a_{i},a_{j}\right)+w_{0}$ is classification prediction model; $\varepsilon_{i}$ is additional noise, with a mean value of zero and a variance of $\sigma^{2}$, where $\varepsilon_{i}\sim N\left(0,\sigma^{2}\right),t_{i}\sim N\left(b_{i},\sigma^{2}\right)$. Assuming that the training sample sets are independent and identically distributed, the observation of vector t obeys the following distribution:

(6) $$m\left(t|a,c,\sigma^{2}\right)={\left(2\pi \sigma^{2}\right)}^{-N/2}\text{exp}[-\frac{1}{2\sigma^{2}}{\left|\left|t-\partial c\right|\right|}^{2}]$$

where ∂ is defined as follows:

(7) $$\partial =\left(\begin{array}{ccc} 1 & k\left(a_{1},a_{1}\right)\cdots & k(a_{1},a_{N})\\ \ldots & \ldots & \ldots \\ 1 & k\left(a_{N},a_{1}\right)\ldots & k\left(a_{N},a_{N}\right) \end{array}\right)$$

The RVM uses sample label t to predict the label $t_{*}$ of the testing sample, given by

(8) $$m\left(t_{*}|t\right)=\int^{\text{​}}p\left(t_{*}|c,\sigma^{2}\right)p(c,\sigma^{2}|t)dwd\sigma^{2}$$

In order to make the value of most components of the weight vector w zero and reduce the computational work of the kernel function, the weight vector w is subjected to additional conditions. Assuming that $c_{i}$ obeys a distribution with a mean value of zero and a variance of $x_{i}^{-1}$, the mean $c_{i}\sim N\left(0,\;x_{i}^{-1}\right)$, $p\left(w|x\right)=\overset{N}{\underset{i=0}{\prod }}p(c_{i}|x_{i})$, where x is a hyper-parameters vector of the prior distribution of the weight vector c.

(9) $$m\left(t_{*}|t\right)=\int^{\text{​}}p\left(t_{*}|w,x,\sigma^{2}\right)p(c,x,\sigma^{2}|t)dwdad\sigma^{2}$$

(10) $$m\left(t_{*}|c,x,\sigma^{2}\right)=N(t_{*}|b\left(a_{*};c\right),\sigma^{2})$$

Because $p\left(c,x,\sigma^{2}|t\right)$ cannot be obtained by an integral, it must be resolved using a Bayesian formula, given by

(11) $$m\left(c,x,\sigma^{2}|t\right)=p\left(x|x,\sigma^{2},t\right)p(x,\sigma^{2}|t)$$

(12) $$m\left(c|x,\sigma^{2},t\right)=p\left(t|c,\sigma^{2}\right)p\left(c|x\right)/p\left(t|x,\sigma^{2}\right)$$

The integral of the product of $p(t|x,\sigma^{2})$ and p(c|x) is given by

(13) $$m\left(t|x,\sigma^{2}\right)={\left(2\pi \right)}^{-N/2}{\left|\Omega \right|}^{-1/2}\text{exp}\left(-\frac{t^{T}\Omega^{-1}t}{2}\right)$$

(14) $$\Omega =\sigma^{2}I+\partial A^{-1}\partial^{T},\;A=diag\left(x_{0,}x_{1,}\ldots ,x_{N}\right)$$

(15) $$m\left(c|x,\sigma^{2},t\right)={\left(2\pi \right)}^{-\left(N+1\right)/2}{\left|\Sigma \right|}^{-1/2}\text{exp}\left(-\frac{{\left(c-u\right)}^{T}\left(c-u\right)}{2}\right)$$

(16) $$\Sigma ={\left(\sigma^{-2}\partial^{T}\partial +A\right)}^{-1}$$

(17) $$u=\sigma^{-2}\Sigma \partial^{T}t$$

Because $\;m(x,\sigma^{2}|t\propto m\left(t|x,\sigma^{2}\right)m\left(x\right)m\left(\sigma^{2}\right)$ and $\;m(x,\sigma^{2}|t$) cannot be solved by means of integration, the solution is approximated using the maximum likelihood method, represented by

(18) $$\left(x_{MP},\sigma_{MP}^{2}\right)=\underset{\;x,\sigma^{2}}{\arg}maxp(t|x,\sigma^{2})$$

The iterative process of $x_{MP}$ and $\sigma_{MP}^{2}$ is as follows:

(19) $$\{\begin{array}{c} x_{i}^{new}=\frac{\gamma_{i}}{\mu_{i}^{2}}\\ {\left(\sigma^{2}\right)}^{new}=\frac{{\left|\left|t-\partial \mu \right|\right|}^{2}}{N-\sum_{i=0}^{N}\mu_{i}}\\ \gamma_{i}=1-x_{i}\sum^{\text{​}}i,i \end{array}$$

where $\sum^{\text{​}}i,i$ is ith element on the diagonal of Σ, and the initial value of a and $\sigma^{2}$ can be determined via the approximation of $a_{MP}$ and $\sigma_{MP}^{2}$ by continuously updating using formula (19). After enough iterations, most of $x_{i}$ will be close to infinity, the value of the corresponding parameters in $c_{i}$ will be zero, and other $x_{i}$ values will be close to finite. The resulting corresponding parameters $a_{i}$ of $x_{i}$ are referred to as the relevance vector now.

### 3.6. Procedure of the Proposed Method

**Figure 2:**
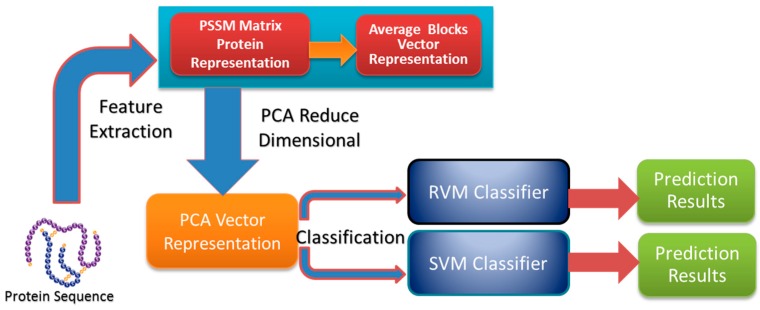
The flow chart of the proposed method.

In the study, the proposed method contains as follows three steps: feature extraction, dimensional reduction using PCA, and sample classification. The feature extraction step contains two steps: (1) a PSSM matrix is used to represent each protein sequence from the datasets; (2) the PSSM matrix of each protein sequence is expressed as a 400-dimensional vector by using the Average Blocks method. Dimensional reduction of the original feature vector is achieved using the PCA method. Finally, sample classification occurs in two steps: (1) the RVM model is employed to implement classification based on the datasets from yeast, *Helicobacter pylori*, *C. elegans*, *M. musculus*, *H. sapiens*, *H. pylori* and *E. coli* whose features have been extracted; (2) the SVM model is used to carry out classification on the yeast dataset. The flow chart of the proposed method is shown in Figure 2.

### 3.7. Performance Evaluation

To evaluate the feasibility and efficiency of the proposed method, four parameters, the accuracy of prediction (Ac), sensitivity (Sn), precision (Pe), and Matthews’ correlation coefficient (Mcc), were computed. They are represented as follows:

(20) $$\text{Ac}=\frac{TP+TN}{TP+FP+TN+FN}$$

(21) $$\text{Sn}=\frac{TP}{TP+FN}$$

(22) $$\text{Pe}=\frac{TP}{FP+TP}$$

(23) $$\text{Mcc}=\frac{\left(TP\times TN\right)-\left(FP\times FN\right)}{\sqrt{\left(TP+FN\right)\times \left(TN+FP\right)\times \left(TP+FP\right)\times \left(TN+FN\right)}}$$

where *TP*, *TN*, *FP*, and *FN* represent true positives, true negatives, false positives, and false negatives, respectively. True positives represent the number of true interacting pairs correctly predicted. True negatives are the number of true non-interacting pairs predicted correctly. False positives stand for the number of true non-interacting pairs falsely predicted, and false negatives are the number of true interacting pairs falsely predicted to be non-interacting pairs. Moreover, we used the Receiver Operating Curve (ROC) to evaluate the performance of our proposed method.

## 4. Conclusions

Although many computational methods have been used to predict PPIs, the effectiveness and robustness of previous prediction models can still be improved. The main objective of this study is to improve prediction accuracy using the proposed approach. In the work, we explore a novel computational method using an RVM classifier combined with Average Blocks and a position specific scoring matrix. From the experimental results, it can be seen that the prediction accuracy of the proposed method is obviously higher than that of previous methods. In addition, our proposed method has also obtained good prediction accuracy on cross-species experiments of five other independent datasets. All these results demonstrated that our proposed method is a very promising and useful support tool for future proteomics research. The main improvements of the proposed method come from adopting an effective feature extraction method that can capture useful evolutionary information. Moreover, the results showed that PCA significantly improves prediction.

## Figures and Tables

**Table 1:** Five-fold cross validation results shown using our proposed method on yeast dataset. Ac: Accuracy; Sn: Sensitivity; Pe: Precision; Mcc: Matthews’s correlation coefficient.

Testing Set	Ac (%)	Sn (%)	Pe (%)	Mcc (%)
1	93.12	92.46	93.64	87.18
2	94.32	94.77	94.02	89.29
3	92.00	92.21	91.96	85.28
4	92.00	91.38	92.14	85.26
5	93.48	93.11	93.94	87.11
Average	92.98 ± 0.99	92.79 ± 1.2	93.14 ± 1.00	86.82 ± 1.66

**Table 2:** Five-fold cross validation results shown using our proposed method on *H. pylori* dataset.

Testing Set	Ac (%)	Sn (%)	Pe (%)	Mcc (%)
1	95.54	97.53	93.56	91.47
2	95.71	94.70	96.95	91.79
3	97.08	97.25	96.92	94.34
4	94.17	94.14	93.45	88.98
5	95.38	94.50	96.69	91.16
Average	95.58 ± 1.0	95.62 ± 1.6	95.51 ± 1.83	91.55 ± 1.91

**Table 3:** Five-fold cross validation results shown using our proposed method on yeast dataset. SVM: Support Vector Machine; PSSM: Position Specific Scoring Matrix; AB: Average Blocks; RVM: Relevance Vector Machine.

Testing Set	Ac (%)	Sn (%)	Pe (%)	Mcc (%)
SVM + PSSM + AB				
-	84.49	84.65	84.27	73.79
-	87.22	88.04	86.81	77.69
-	84.09	84.41	84.11	73.23
-	85.47	85.05	85.12	75.15
-	86.16	85.47	86.63	76.15
Average	85.49 ± 1.26	85.52 ± 1.46	85.39 ± 1.28	75.20 ± 1.80
RVM + PSSM + AB				
-	93.12	92.46	93.77	87.18
-	94.32	94.77	93.86	89.29
-	92.00	92.21	91.79	85.28
-	92.00	91.38	92.59	85.26
-	93.48	93.11	93.86	87.11
Average	92.98 ± 0.99	92.79 ± 1.2	93.14 ± 1.00	86.82 ± 1.66

**Table 4:** Prediction performance on five species based on our model. PPV: Positive Predictive Value; NPV: Negative Predictive value; F1: F-Score.

Testing Set	Ac (%)	Sn (%)	PPV (%)	NPV (%)	F1 (%)
*H. pylori*	91.55	91.55	100%	0	95.59
*M. musculus*	89.4	89.46	100%	0	94.44
*H. sapiens*	91.08	91.08	100%	0	95.33
*E. coli*	94.81	94.81	100%	0	97.34
*C. elegans*	88.31	88.31	100%	0	93.79

**Table 5:** Predicting ability of different methods on the yeast dataset. ACC: Auto Covariance; LD: Local Description; PCA: Principal Component Analysis; EELM: Ensemble Extreme Learning Machines; N/A: No Available.

Model	Testing Set	Ac (%)	Sn (%)	Pe (%)	Mcc (%)
Guos’ work [10]	ACC	89.33 ± 2.67	89.93 ± 3.60	88.77 ± 6.16	N/A
	AC	87.36 ± 1.38	87.30 ± 4.68	87.82 ± 4.33	N/A
Zhous’ work [14]	SVM + LD	88.56 ± 0.33	87.37 ± 0.22	89.50 ± 0.60	77.15 ± 0.68
Yangs’ work [15]	Cod1	75.08 ± 1.13	75.81 ± 1.20	74.75 ± 1.23	N/A
	Cod2	80.04 ± 1.06	76.77 ± 0.69	82.17 ± 1.35	N/A
	Cod3	80.41 ± 0.47	78.14 ± 0.90	81.66 ± 0.99	N/A
	Cod4	86.15 ± 1.17	81.03 ± 1.74	90.24 ± 1.34	N/A
Yous’ work [16]	PCA-EELM	87.00 ± 0.29	86.15 ± 0.43	87.59 ± 0.32	77.36 ± 0.44
Proposed method	RVM	92.98 ± 0.99	92.79 ± 1.2	93.14 ± 1.00	86.82 ± 1.66

**Table 6:** Predicting ability of different methods on the *H. pylori* dataset.

Model	Ac (%)	Sn (%)	Pe (%)	Mcc (%)
Nanni [17]	83	86	85.1	N/A
Nanni [18]	84	86	84	N/A
Nanni and Lumini [6]	86.6	86.7	85	N/A
Z-H You [16]	87.5	88.95	86.15	78.13
L Nanni [18]	84	84	86	N/A
Proposed method	95.58	95.62	95.51	91.55

**Table 7:** Five-fold cross validation results shown using our proposed method on yeast dataset.

Dataset	Testing Set	Ac (%)
The Original Dataset	1	87.66
	2	88.16
	3	87.17
	4	87.84
	5	85.92
The Dataset Processed by Using PCA	1	93.12
	2	94.32
	3	92.00
	4	92.00
	5	93.48
